# Granzyme A Is Required for Regulatory T-Cell Mediated Prevention of Gastrointestinal Graft-versus-Host Disease

**DOI:** 10.1371/journal.pone.0124927

**Published:** 2015-04-30

**Authors:** Sarvari Velaga, Sya N. Ukena, Ulrike Dringenberg, Christina Alter, Julian Pardo, Olivia Kershaw, Anke Franzke

**Affiliations:** 1 Department of Hematology, Hemostasis, Oncology and Stem Cell Transplantation, Hannover Medical School, Hannover, Germany; 2 Immune Effector Cells Group (ICE), Biomedical Research Centre of Aragon (CIBA), Zaragoza, Spain; 3 Department of Veterinary Pathology, Freie Universität Berlin, Berlin, Germany; The Jackson Laboratory for Genomic Medicine, UNITED STATES

## Abstract

In our previous work we could identify defects in human regulatory T cells (Tregs) likely favoring the development of graft-versus-host disease (GvHD) following allogeneic stem cell transplantation (SCT). Treg transcriptome analyses comparing GvHD and immune tolerant patients uncovered regulated gene transcripts highly relevant for Treg cell function. Moreover, granzyme A (GZMA) also showed a significant lower expression at the protein level in Tregs of GvHD patients. GZMA induces cytolysis in a perforin-dependent, FAS-FASL independent manner and represents a cell-contact dependent mechanism for Tregs to control immune responses. We therefore analyzed the functional role of GZMA in a murine standard model for GvHD. For this purpose, adoptively transferred CD4^+^CD25^+^ Tregs from *gzmA* ^-/-^ mice were analyzed in comparison to their wild type counterparts for their capability to prevent murine GvHD. *GzmA* ^-/-^ Tregs home efficiently to secondary lymphoid organs and do not show phenotypic alterations with respect to activation and migration properties to inflammatory sites. Whereas *gzmA* ^-/-^ Tregs are highly suppressive *in vitro*, Tregs require GZMA to rescue hosts from murine GvHD, especially regarding gastrointestinal target organ damage. We herewith identify GZMA as critical effector molecule of human Treg function for gastrointestinal immune response in an experimental GvHD model.

## Introduction

Regulatory T cells (Tregs) play a critical role in the induction and maintenance of peripheral tolerance in allogeneic stem cell transplantation (SCT). Experiments in graft-versus-host disease (GvHD) model systems have shown that removal of CD4^+^CD25^+^ Tregs from the donor allograft accelerates GvHD, whereas the adoptive transfer of Tregs inhibits the allogeneic immune response [1–4]. Recently, SCT qualified as first venue for donor Treg cell infusion and revealed promising results [5]. Especially in HLA-haploidentical SCT coinfusion of regulatory and conventional T cells protects against GvHD and prevents acute leukemia relapse in high risk patients [6]. In the last decade, surface molecules, transcription factors, and soluble molecules have been identified that are highly important for the induction of *in vivo* tolerance by Tregs [7]. As functional data for regulatory T cells are very rare, our recent data studying the human Treg transcriptome following allogeneic SCT are highly relevant [8]. This comparative analysis in more than 140 patients with and without GvHD gives a global view on immune homeostasis of Tregs in the allogeneic setting. We identified several key molecules likely responsible for defective Treg function in GvHD patients with regards to their suppressive capacity (i.e., GZMA) and migration to inflammatory sites (i.e., CXCR3, CCR5). Tregs of GvHD patients show a significant lower expression of GZMA early after SCT in comparison to immune tolerant patients never developing a GvHD, but stable expression levels of granzyme B (GZMB). Thereby, our results are well in line with murine data demonstrating that GZMB is not required for Treg cell mediated suppression of GvHD [9]. Notably, to our knowledge the functional role of GZMA has not been tested for Treg cell mediated GvHD prevention. The proposed functions of granzymes are multifaceted including induction of cell death and inflammation [10]. Several groups demonstrated that human Tregs can use the granzyme/ perforin pathway to suppress effector T cell proliferation and effectively kill autologous immune cells including activated CD4^+^ and CD8^+^ T cells and dendritic cells [11–13]. GZMA is the most abundant serine protease that has been proposed to induce a caspase-independent cell death in the target cells [14]. With respect to our data from human Treg transcriptome study [8], we here examined the role of GZMA in a haploidentical murine GvHD model using *gzmA* ^*-/-*^ donor Tregs to clarify the functional relevance of GZMA for Treg-mediated suppression of GvHD.

## Material and Methods

### Information on animal experiments

The animal experiments were performed in accordance to the guidelines and approval by Niedersächsisches Landesamt für Verbraucherschutz und Lebensmittelsicherheit (Application number: 33.9-42502-04-09/1644). All efforts were made to prevent animal suffering. In addition, mice numbers were kept as small as necessary for appropriate statistical analyses. During the experiments mice were monitored twice daily for any signs of pain and distress according to the Cooke Score, which includes parameters as activity, weight loss and posture (see also description of the GvHD model later in this section). The optimal irradiation dose has been titrated to the lowest possible dose of 8 Gy during the establishment of the GvHD model at the Hannover Medical School according to the animal research application (see above). To minimize suffering of animals mice were sacrified latest after 4 weeks by cervical dislocation. Notably, experiments were discontinued at an earlier timepoint for animals with a body weight loss of more than 20% and a Cooke score of more than 10. *gzmA* ^-/-^ mice were kindly provided by Julian Pardo, Immune Effector Cells Group (ICE), Aragón Health Research Institute (IIS Aragón), Edificio CIBA, Biomedical Research Centre of Aragon (CIBA), Zaragoza. Cell populations used in the experiments within this manuscript have been freshly isolated from mice. No cell lines or human tissue have been used.

### Treg isolation and phenotypic characterization

CD4^+^ T cells from wildtype (WT) and *gzmA* ^-/-^ mice (the genotype was analyzed as described elsewhere [15,16]) were enriched from splenocytes using CD4^+^ T cell isolation kit (Miltenyi Biotech, Germany). Negative selection of CD4^+^CD25^+^ Tregs and positive enrichment of CD4^+^CD25^-^ T effector cells from splenocytes was performed using mouse CD4^+^25^+^ regulatory T cell isolation kit (Miltenyi Biotech, Germany). Total CD4^+^25^+^ Treg cells isolated from peripheral lymph nodes and spleens of WT and *gzmA* ^-/-^ mice were stained with anti-mouse CD4 FITC, anti-mouse CD25 PE, anti-mouse CD44 APC-Cy7, anti-mouse ICOS A647, anti-mouse CD62L PerCp and anti-mouse CXCR3 Pe-Cy7, measured by FACS Canto and analyzed and calculated using FloJo Software (gating strategies are presented in S1 and S2 Figs).

### Inhibition of proliferation

The suppressive capacity of WT and *gzmA* ^-/-^ Tregs was determined as described earlier [17]. Briefly, 5x10^4^ autologous CD4^+^CD25^-^ T responder (Tresp) cells were stained with 0.5 μM CFSE (Invitrogen) according to the manufacturer’s protocol. CFSE-stained Tresp were activated with αCD3/αCD28 mouse Dynabeads (Gibco) and cultured in the presence of WT or *gzmA* ^-/-^ Tregs isolated from spleen in RPMI 1640 containing Glutamax medium (Gibco) supplemented with 10% FCS (Biochrom), 50 μg/ml gentamycin (Sigma Aldrich) and 50 μg/ml penicillin/streptomycin (Sigma Aldrich) at ratios of 1:2, 1:1, 2:1 and 4:1. After seven days cells were analyzed by FACS Canto (BD). Percentage of suppression was calculated as followed: 100—(percentage of proliferated Tresp x 100 / percentage of proliferated Tresp).

### Adoptive transfer of Tregs in a GvHD mouse model

The standard model for acute GvHD (C57BL/6 (H-2K^b^) → BALB/c (H-2K^d^)) [3] with a total body irradiation of 800cGy followed by i.v. tail injection of T cell depleted bone marrow (TCD BM) cells followed by adoptive transfer of effector T cells with and without Tregs for determination of *in vivo* effects. In more detail, as stem cell source for transplantation enriched bone marrow cells were isolated from WT mice using the CD90.2 microbeads (Miltenyi Biotech, Germany). CD4^+^ T cells were enriched from splenocytes using CD4^+^ T cell isolation kit (Miltenyi Biotech, Germany). Negative selection of WT and *gzmA* ^-/-^ CD4^+^CD25^+^ Tregs and positive enrichment of WT CD4^+^CD25^-^ T effector cells from splenocytes was performed using mouse CD4^+^25^+^ regulatory T cell isolation kit (Miltenyi Biotech, Germany). Purity of all T cell populations were assessed by flow cytometry using antibodies against CD4, CD25, CD19 (marker for B cells), CD8 (marker for cytotoxic T cells), CD11c (marker for dendritic cells) and erythrocyte marker Gr1. Following isolation and FACS analyses TCD BM and isolated T cell populations of C57/BL6 (WT) and *gzmA* ^*-/-*^ donor mice (purity > 95%), respectively were transferred into BALB/c recipient mice after lethal irradiation. Mice receiving TCD BM (5x10^6^), WT CD4^+^CD25^-^ Teff and WT Tregs (each 0.25x10^6^) represented the immune tolerant control group (group A, n = 3), whereas recipients with adoptively transferred TCD BM (5x10^6^) and WT CD4^+^CD25^-^ Teff (0.25x10^6^) without Treg transfer represented the GvHD group (group B, n = 3). Group C (n = 3) comprised mice receiving TCD BM (5x10^6^) + WT CD4^+^CD25^-^ Teff and *gzmA* ^*-/-*^ Tregs (each 0.25x10^6^). After adoptive T cell transfer the incidence, severity and clinical manifestation of GvHD was monitored comparatively in the 3 groups by clinical and histopathological grading. The clinical GvHD scoring was performed for weight loss, posture, activity, fur texture and skin integrity from 0–10 according to Cooke and colleagues [18]. For histopathological investigations and scoring, the spleen as well as the GvHD target organs liver, small and large intestine of sacrificed mice were collected after 3 weeks and analyzed in a blinded manner at the Faculty of Veterinary Medicine, Department of Veterinary Pathology (Freie Universität Berlin, Berlin, Germany).

## Results

### Phenotypic characterization of *gzmA* ^*-/-*^ Tregs efficiently homing to secondary lymphoid organs

**Fig 1:**
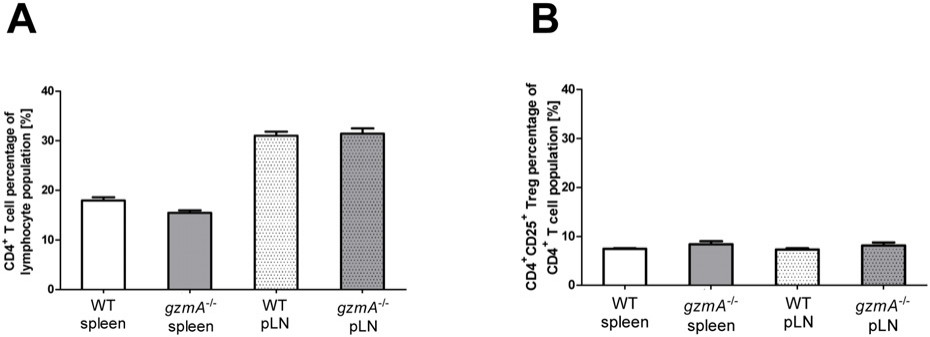
*GzmA* ^*-/-*^ Tregs home efficiently to secondary lymphoid organs. CD4^+^ (A) and CD4^+^CD25^+^ Tregs (B) were isolated from spleen and peripheral lymph node from wildtype (WT; n = 3) and *gzmA* ^*-/-*^ mice (n = 3) and analyzed by FACS. Expression values were calculated as a percentage of the T lymphocyte population and mean values are presented as bar graphs. Students t-test did not reveal any significant differences between WT and *gzmA* ^*-/-*^ Tregs.

**Fig 2:**
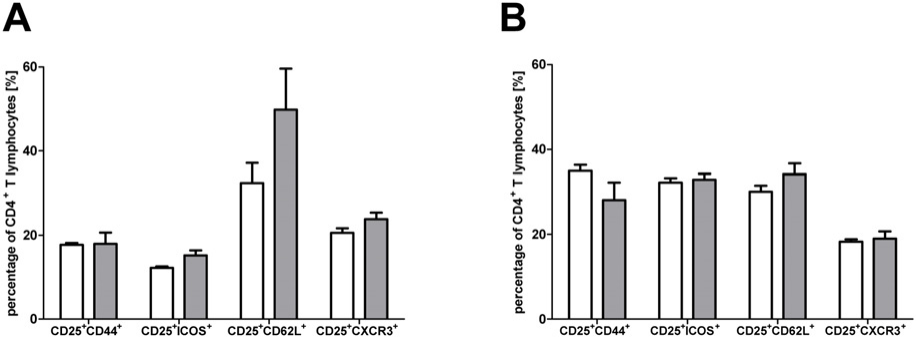
*GzmA* ^*-/-*^ Tregs show no phenotypic alteration with respect to activation and migration. WT Tregs (white bars) and *gzmA* ^*-/-*^ Tregs (grey bars) were isolated from spleen (A) and peripheral lymph nodes (B) and analyzed by FACS. Expression values were calculated as a percentage of the CD4^+^ T lymphocyte population and mean values are presented as bar graphs. Students t-test did not reveal any significant differences between WT and *gzmA* ^*-/-*^ Tregs. Results are shown for n = 3 WT and *gzmA* ^*-/-*^ mice each.

Wild type (WT) mice and *gzmA* ^-/-^ mice were compared for Treg cell number and expression of surface marker differentiating naïve, activated and memory T cells (CD62L, CD44). Furthermore, the expression of CXCR3 was analyzed on the respective CD4^+^CD25^+^ Treg cell population in order to characterize the properties of Tregs to migrate to Th1-associated inflammatory sites. For this purpose immune cells were prepared from spleens and peripheral lymph nodes and comparatively analyzed by FACS. Absolute Treg numbers in spleen and peripheral lymph nodes from WT and *gzmA* ^-/-^ mice did not show significant differences. CD4^+^ T cells encounter 31,0% and 31,4% of the lymphocyte population in the peripheral lymph nodes of WT and *gzmA* ^-/-^ mice respectively, but only 17,9% and 15,4% in the spleen (Fig 1A). In contrast, the percentage of CD4^+^CD25^+^ T cells within the lymphocyte populations in peripheral lymph nodes and spleen are comparable without differences between WT and *gzmA* ^-/-^ mice (7,3% versus 8,1% and 7,4% versus 8,4%) (Fig 1B). FACS analyses of Tregs from spleen and peripheral lymph nodes did not reveal any significant differences. A comparable proportion of CD25^+^ Tregs in spleen (20,6% and 23,9%) and lymph nodes (18,3% and 19,0%) of WT and *gzmA* ^-/-^ mice express the Th1-associated chemokine receptor CXCR3 enabling Tregs to migrate to inflammatory sites like GvHD target organs. More interestingly, although not significant, we detected a higher proportion of CD62L^+^ expressing CD25^+^ T cells in the spleen of *gzmA* ^*-/-*^ mice compared to WT mice (Fig 2A).

### GZMA expressing Tregs protect against GvHD-related tissue damage of the intestine

**Fig 3:**
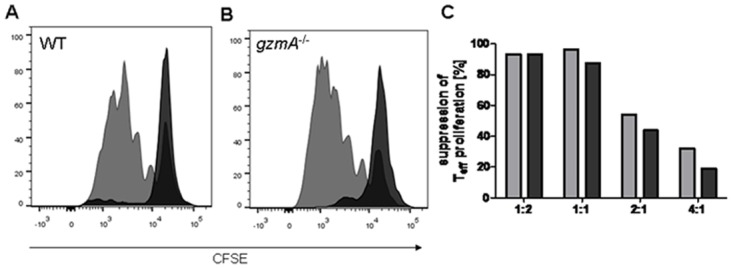
*GzmA* ^*-/-*^ Tregs are highly suppressive *in vitro*. Regulatory T cells isolated from WT (A) or *gzmA* ^*-/-*^ (B) mice were co-cultured with CD4^+^CD25^-^ T responder cells and activated with αCD3/αCD28. The proliferation of the responder cells was measured by the dilution of the proliferation dye CFSE. (A) and (B) show the proliferative response with or without (grey) regulatory T cells in a ratio 1:1. Percentage of suppression is shown for WT and *gzmA* ^*-/-*^ Tregs in different effector T cell to regulatory T cell ratios (C).

**Fig 4:**
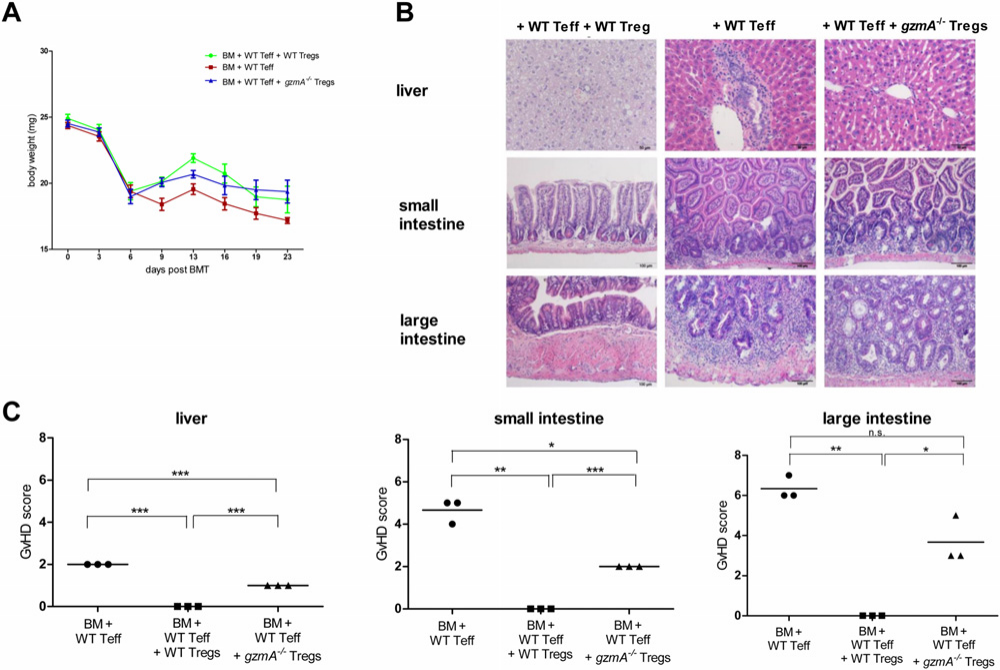
*GzmA* ^*-/-*^ Tregs are required for prevention of gastrointestinal GvHD. Lethally irradiated BALB/c mice (n = 9) received T cell depleted (TCD) bone marrow cells (BM) together with CD4^+^CD25^-^ T effector cells (squares, n = 3) and CD4^+^CD25^+^ WT Treg cells (circle, n = 3) or CD4^+^CD25^+^ *gzmA* ^*-/-*^ Treg cells (triangle, n = 3) from WT mice. (A) Body weight of the recipients was monitored daily. (B) Histopathological investigations of liver, small and large intestine of mice sacrified after 3 weeks. (C) Histopathological GvHD scoring 3 weeks after transplantation were graded for severity of inflammation and degree of organ destruction resulting in an organ-specific cumulative pathology score. For statistical analysis student`s t-test was performed and p values <0.05 (*) were considered significant, while p<0.01 (**) and p<0.001 (**) were considered highly significant.

Interestingly, results from the *in vitro* proliferation-/inhibition assay show that GZMA is not required for the direct inhibition of effector T cell proliferation (Fig 3). However, the more complex role of GZMA in Treg cell mediated suppression *in vivo* was analyzed in a standard model of acute GvHD. For this purpose, T cell depleted bone marrow cells (TCD BM) and isolated T cell populations of wild type (WT) and *gzmA* ^*-/-*^ C57/BL6 donor mice were transferred into the BALB/c recipient mice after lethal irradiation. Immune tolerant (TCD BM + Teff + WT Tregs or *gzmA* ^*-/-*^ Tregs) and GvHD mice (TCD BM + Teff) were monitored comparatively after adoptive T cell transfer for incidence, severity and clinical manifestation of GvHD by clinical and histopathological grading (see Materials and Methods). After irradiation and transplantation the body weight of recipient mice declined in all treatment groups and increased again one week after transplantation (Fig 4A). However, two weeks after transplantation, mice from the GvHD group continuously lost weight in contrast to the immune tolerant groups. Histopathological investigations confirm a severe inflammation especially in the small and large intestine as GvHD target organs (Fig 4B). Recipients from the immune tolerant group receiving WT Tregs recovered quickly and showed the highest body weight, whereas mice after adoptive transfer of *gzmA* ^*-/-*^ Tregs recovered slower and reached a body weight similar to the immune tolerant mice receiving WT Tregs after 3 weeks with only limited/moderate inflammation of GvHD target organs (Fig 4A). Moreover, clinical GvHD grading of mice receiving WT Tregs did not show any signs of inflammation or organ destruction in the liver and intestinal tract three weeks after transplantation resulting in a pathology scoring of “0” for each respective target organ (Fig 4C). In contrast, mice of the GvHD group developed a severe cellular inflammation as expected going along with organ destruction in form of crypt apoptosis in the intestinal tract (cumulative mean pathology score of the small and large intestine: “4.8” and “6.2”) and bile duct injury (mean pathology score of “2”). Notably, the adoptive transfer of *gzmA* ^*-/-*^ Treg could not prevent GvHD efficiently. However, in the liver the degree of inflammation and bile duct destruction was only mild (mean pathology score “1”) and in the GvHD group significant severe GvHD-related signs could be observed (p<0.001). In contrast, *gzmA* ^*-/-*^ Tregs could not efficiently protect from GvHD-related inflammation and organ destruction in the intestinal tract with relevant crypt apoptosis detected in the small (mean pathology score “2” and large intestine (mean pathology score “3”) (Fig 4C).

## Discussion

Recently, we have reported that Tregs of GvHD patients may not effectively control alloreactive immune responses due to defects with respect to suppressive/cytolytic capacities, migration properties and clonal expansion [8]. Tregs of GvHD patients show a significant lower expression of GZMA early after SCT in comparison to immune tolerant patients never developing a GvHD, but stable expression levels of granzyme B (GZMB). Whereas others could demonstrate that GZMB is not required for Treg cell mediated suppression of murine GvHD [9] and thereby supporting our results in patients after allogeneic SCT, confirmative functional analyses of GZMA are still missing. The ability of donor-type WT Treg cells to protect from acute GvHD is well known [2–4,19]. In this report we now demonstrate that GZMA is a highly relevant effector molecule of Tregs for the prevention of GvHD, especially of the intestinal tract as target organ. Our phenotypic analysis of *gzmA* ^*-/-*^ Tregs revealed an increased but not significant expression of the lymphoid homing receptor CD62L (L-selectin) on *gzmA* ^*-/-*^ Tregs compared to WT Tregs in the spleen probably indicating a more efficient homing of *gzmA* ^*-/-*^ Tregs to secondary lymphoid tissues. Expression of CD62L^+^CD4^+^CD25^+^ Tregs has been described to be pivotal for efficient migration of Tregs to priming sites of allogeneic immune responses. Only CD62L^+^CD4^+^CD25^+^ Tregs prevent severe tissue damage to the colon and protected from lethal GvHD [20] by inhibition of the expansion of donor derived activated CD4^+^CD25^-^ T cells. However, we could not detect a more efficient homing of *gzmA* ^*-/-*^ Tregs to secondary lymphoid homing and their migration capacity and suppressive function is comparable to WT Tregs. Therefore, additional factors *in vivo* might be responsible for the protection from inflammation and crypt destruction in the small and especially large intestine by GZMA and not by GZMB [9] expressing Tregs. Interestingly, upon activation CD4^+^CD25^+^ natural Tregs predominantly express GZMA, while GZMB seems not to be inducible [13]. GZMA expressing Tregs may be required to suppress/kill activated autologous immune cells that express endogenous inhibitors of GZMB, such as serin proteinase inhibitor PI-9 [21]. Furthermore, Grossman et al. could show that GZMA expressing Tregs exhibit a more effective cytotoxicity and were able to kill autologous immune cells at a very low E:T ratio in comparison to GZMB expressing Tregs [12]. The induction of apoptosis is not restricted to effector T cells but also involves APCs. In this respect, human Tregs were found to induce apoptosis in B cells, monocytes, and DCs, thereby mediating indirect regulation of T-cell responses [12]. Furthermore, GZMA can cleave a number of extra cellular matrix proteins, and thus may facilitate migration of Tregs through extracellular tissues. In contrast, GZMB has been shown to be required not only for cytolytic regulation of lung inflammation by Tregs during acute viral lung infection [22] as well as for Treg cell-contact dependent mediated inhibition of effector T cell function in tumor immunity [23] and transplantation [24], but not for protection from GvHD [9]. Similar to WT Tregs the adoptive transfer of *gzmB* ^*-/-*^ Tregs protected from lethal GvHD and organ damage. Differences regarding the impact of GZMA and GZMB in GvHD prevention, especially for Treg cell mediated protection from intestinal GvHD, might therefore be multifaceted and dependent on environmental factors of the gastrointestinal tract. As also our human data support the high impact of GZMA in Treg cell mediated protection from GvHD following allogeneic stem cell transplantation further dissection of the molecular mechanism is warranted.

## Supporting Information

S1 Fig Gating strategies for analyses of CD4^+^ and CD4^+^CD25^+^ T cell populations. CD4^+^ and CD4^+^CD25^+^ Tregs were isolated from spleen and peripheral lymph node from wildtype (WT) and *gzmA* ^*-/-*^ mice by MACS separation and stained with respective anti-mouse antibodies for measurement by FACS Canto. Representative data of one WT and one *gzmA* ^*-/-*^ mice are shown.
(TIF)

S2 Fig Gating strategies for analyses of indicated T cell populations. Total CD4^+^25^+^ Treg cells isolated from peripheral lymph nodes and spleens of WT and *gzmA* ^-/-^ mice were stained with respective anti-mouse antibodies for measurement by FACS Canto. Representative data of one WT and one *gzmA-/-* mice are shown.
(TIF)
