# Supplementary figures and images for: Granzyme A Is Required for Regulatory T-Cell Mediated Prevention of Gastrointestinal Graft-versus-Host Disease
Source: PLoS One. 2015 Apr 30;10(4):e0124927. doi: 10.1371/journal.pone.0124927 (PMC4415808; doi:10.1371/journal.pone.0124927)

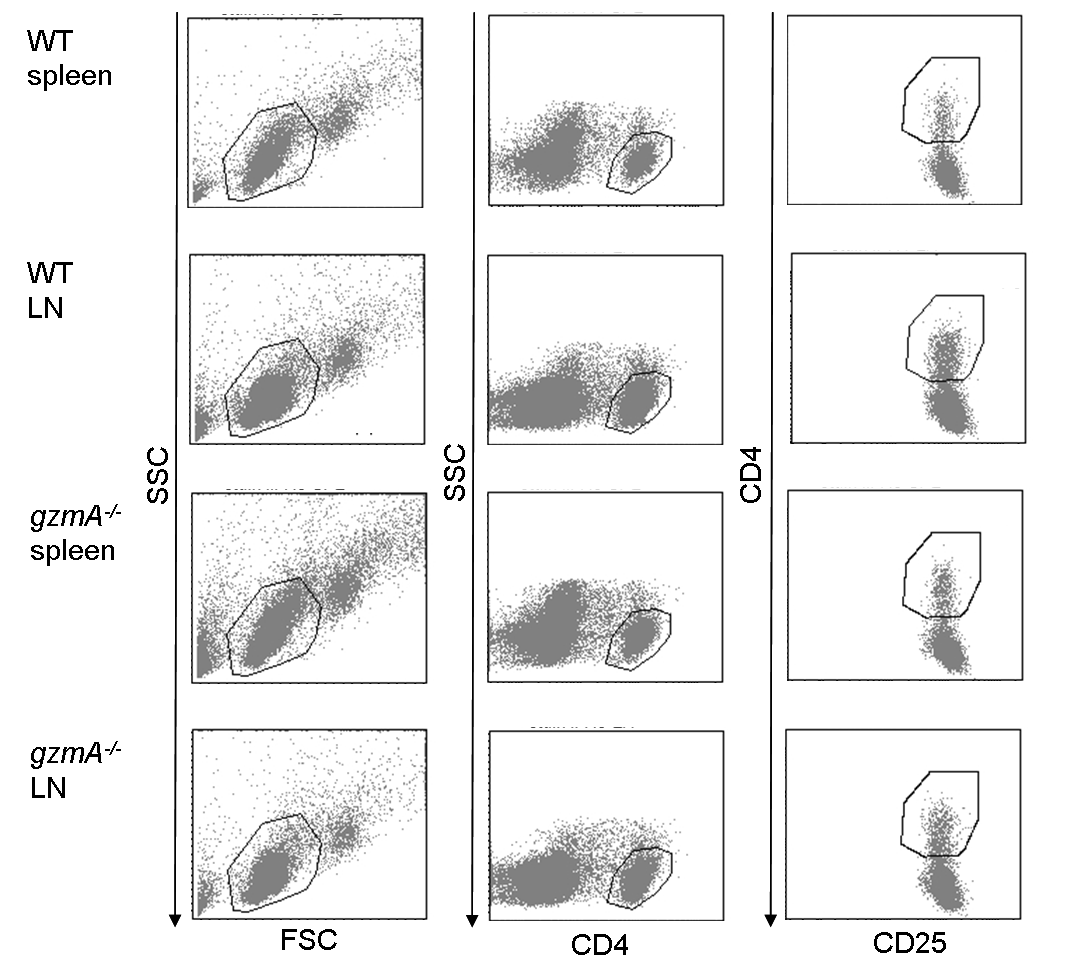

Supplement: S1 Fig — CD4+ and CD4+CD25+ Tregs were isolated from spleen and peripheral lymph node from wildtype (WT) and gzmA -/- mice by MACS separation and stained with respective anti-mouse antibodies for measurement by FACS Canto. Representative data of one WT and one gzmA -/- mice are shown. (TIF) [file pone.0124927.s001.tif]

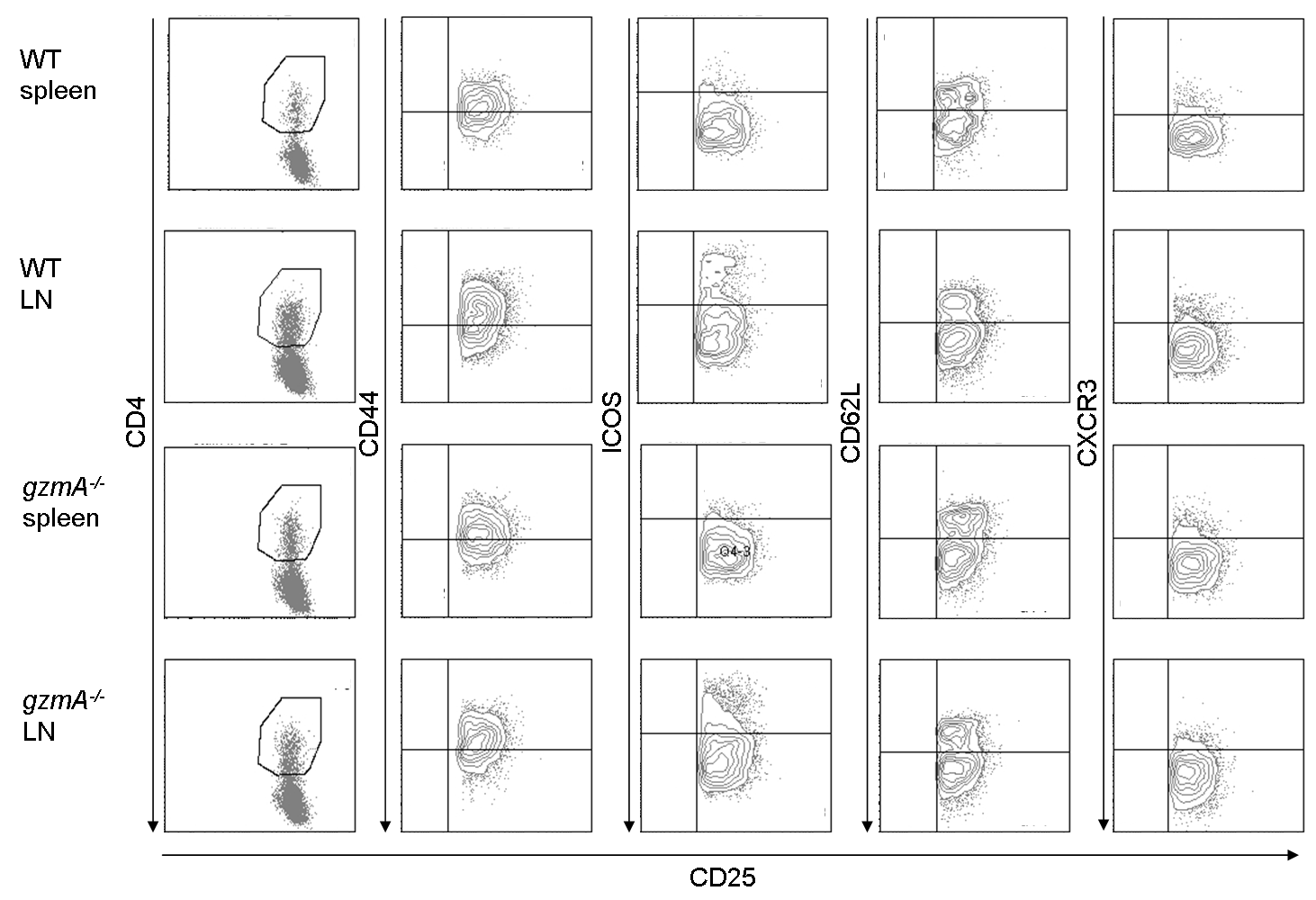

Supplement: S2 Fig — Total CD4+25+ Treg cells isolated from peripheral lymph nodes and spleens of WT and gzmA -/- mice were stained with respective anti-mouse antibodies for measurement by FACS Canto. Representative data of one WT and one gzmA-/- mice are shown. (TIF) [file pone.0124927.s002.tif]
